# Aberrant accumulation of NIK promotes tumor growth by dysregulating translation and post-translational modifications in breast cancer

**DOI:** 10.1186/s12935-023-02904-y

**Published:** 2023-04-01

**Authors:** Yusuke Hayashi, Jun Nakayama, Mizuki Yamamoto, Masashi Maekawa, Shinya Watanabe, Shigeki Higashiyama, Jun-ichiro Inoue, Yusuke Yamamoto, Kentaro Semba

**Affiliations:** 1 Department of Life Science and Medical Bioscience, School of Advanced Science and Engineering, Waseda University, TWIns, 2-2 Wakamatsu-Cho, Shinjuku-Ku, Tokyo, 162-8480 Japan; 2 Laboratory of Integrative Oncology, National Cancer Center Research Institute, 5-1-1 Tsukiji, Chuo-Ku, Tokyo, 104-0045 Japan; 3 Research Center for Asian Infectious Diseases, The Institute of Medical Science, The University of Tokyo, Shirokane-Dai, Minato-Ku, Tokyo, 108-8639 Japan; 4 Division of Cell Growth and Tumor Regulation, Proteo-Science Center, Ehime University, Toon, 791-0295 Japan; 5 Department of Biochemistry and Molecular Genetics, Ehime University Graduate School of Medicine, Toon, 791-0295 Japan; 6 Division of Physiological Chemistry and Metabolism, Graduate School of Pharmaceutical Sciences, Keio University, Minato-Ku, Tokyo, 105-8512 Japan; 7 Translational Research Center, Fukushima Medical University, Fukushima, 960-1295 Japan; 8 Department of Molecular and Cellular Biology, Osaka International Cancer Institute, Chuo-Ku, Osaka, 541-8567 Japan; 9 Research Platform Office, The Institute of Medical Science, The University of Tokyo, Shirokane-Dai, Minato-Ku, Tokyo, 108-8639 Japan

**Keywords:** NIK, Non-canonical NF-κB, Translation, Post-translational regulation, In vivo selection, Orthotopic xenograft, Breast cancer

## Abstract

**Background:**

In vivo investigations with cancer cells have powerful tools to discover cancer progression mechanisms and preclinical candidate drugs. Among these in vivo experimental models, the establishment of highly malignancy cell lines with xenograft has been frequently used. However, few previous researches targeted malignancy-related genes whose protein levels translationally changed. Therefore, this study aimed to identify malignancy-related genes which contributed to cancer progression and changed at the protein level in the in vivo selected cancer cell lines.

**Methods:**

We established the high malignancy breast cancer cell line (LM05) by orthotopic xenograft as an in vivo selection method. To explore the altered genes by translational or post-translational regulation, we analyzed the protein production by western blotting in the highly malignant breast cancer cell line. Functional analyses of the altered genes were performed by in vitro and in vivo experiments. To reveal the molecular mechanisms of the regulation with protein level, we evaluated post-translational modification by immunoprecipitation. In addition, we evaluated translational production by click reaction-based purification of nascent protein.

**Results:**

As a result, NF-κB inducing kinase (NIK) increased at the protein level and promoted the nuclear localization of NF-κB2 (p52) and RelB in the highly malignant breast cancer cell line. The functional analyses indicated the NIK upregulation contributed to tumor malignancy via cancer-associated fibroblasts (CAFs) attraction and partially anti-apoptotic activities. Additionally, the immunoprecipitation experiment revealed that the ubiquitination of NIK decreased in LM05 cells. The decline in NIK ubiquitination was attributed to the translational downregulation of cIAP1.

**Conclusions:**

Our study identified a dysregulated mechanism of NIK production by the suppression of NIK post-modification and cIAP1 translation. The aberrant NIK accumulation promoted tumor growth in the highly malignant breast cancer cell line.

**Supplementary Information:**

The online version contains supplementary material available at 10.1186/s12935-023-02904-y.

## Background

Breast cancer is the most common cancer among women worldwide [1]. Although breast cancer mortality has decreased for three decades since the 1990s, the decreasing trend in mortality has slowed in recent years [1, 2]. Breast cancer has been traditionally categorized into four molecular subtypes (luminal A, luminal B, human epidermal growth factor receptor type 2 (HER2) -enriched, and triple-negative) based on the expression of pathological marker proteins such as HER2, estrogen receptor (ER), and progesterone receptor (PR). In particular, triple-negative breast cancer (TNBC) constitutes approximately 10% ~ 20% of breast cancers and is characterized by defects in HER2, ER, and PR expression [3, 4]. TNBC presents a relatively poor prognosis, with metastases occurring more frequently than in other subtypes despite the limited molecular therapeutic targets [3, 5]. Therefore, it is necessary to explore innovative therapeutic targets by further elucidating the malignant mechanism of TNBC.

In normal cells, translational and post-translational modification tightly regulate signal transductions, while, in cancer cells, abnormalities in translational and post-translational modifications contribute to their malignancy [6, 7]. During the translation process, oncogenic signaling activation, such as through the mTOR and RAS-MAPK pathways, induces an enhancement in eIF4F complex expression and phosphorylation, thereby promoting global translation and contributing to cancer malignancy [6]. On the other hand, unusual tRNA modification and RNA conformations also contribute to cancer progression by promoting the translation of specific genes [8–10]. Post-translational modifications involve a large variety of mechanisms. For example, ubiquitination, one of the major post-translational modifications, has a large number of regulatory functions, including proteostasis, signaling complex assembly, chromatin remodeling, and protein secretion [7]. In particular, protein degradation by ubiquitination is essential for the regulation of signaling pathways, such as MAPK, NF-κB and PI3K-AKT-mTOR, which play important roles in cell growth and survival [7]. Disruption of ubiquitin-modifying machinery can lead to the malignant transformation of cancer and a variety of other diseases [7, 11]. To elucidate mechanisms of cancer malignancy, transcriptome analysis is frequently used, and the development of analysis at single-cell level has made it possible to conduct more detailed analysis [12]. However, it is also necessary to conduct analyses integrating transcriptome analysis, translational and post-translational modification evaluations at the protein level.

In vivo investigations with cancer cell line-derived models have contributed to the understanding of cancer biology and cancer hallmarks [13]. Animal models enable to analyze the functions of certain genes involved in cancer malignancy and the evaluation of the antitumor properties of preclinical candidate drugs. Among these models, xenograft models have been frequently utilized to assess tumor growth at particular organs and metastases to distant organs. Xenograft models are classified into two categories: orthotopic and ectopic. In particular, an orthotopic xenograft (OX) model, which mimics early cancer progression, is appropriate to comprehensively understand tumorigenesis and metastatic mechanisms. We previously established a cell line, LM05, with high tumor growth and lung-metastatic properties from a TNBC cell line MDA-MB-231 using an OX model [14]. Interestingly, this cell line showed a different expression profile from that of another lung-metastatic cell line, LM1-2–1, established by tail vein injection (TVI) that corresponds to an ectopic xenograft model [14, 15]. However, the molecular mechanisms of high tumor growth and lung-metastatic properties of LM05 cells remain unclear.

In this study, we comparatively analyzed western blot and microarray data of LM05 cells to discover their specific activating signals in comparison with parental MDA-MB-231 cells and LM1-2-1 cells. As a result, we identified that the nuclear localization of NF-κB2 (p52) and RelB, classified as non-canonical NF-κB pathway, was facilitated in LM05 cells via NIK upregulation at the protein level. The aberrant accumulation of NIK due to translational downregulation of cIAP1 promoted tumor growth via a cancer-inducing inflammatory response.

## Methods

### Cell culture

MDA-MB-231-*mSlc7a1*-*luc2* (parental cells), LM05 and LM1-2-1 cells were established as previously described [14]. These cell lines were cultured in RPMI-1640 (Fujifilm Wako Pure Chemical Corporation, Osaka, Japan) supplemented with 10% heat-inactivated FBS (Nichirei Biosciences Inc., Tokyo, Japan), 100 U/mL penicillin (Meiji Seika Pharma Co., Ltd., Tokyo, Japan) and 100 μg/mL streptomycin (Meiji-Seika Pharma) at 37 °C in 5% CO_2_. Plat-E packaging cells were kindly provided from T. Kitamura (Institute of Medical Science, University of Tokyo), and TIG-3 cells were kindly provided from Dr. B. Shiotani (National Cancer Center Research Institute). These cells were cultured in DMEM (Fujifilm Wako Pure Chemical Corporation) supplemented with 10% heat-inactivated FBS, 100 U/mL penicillin, and 100 μg/mL streptomycin. MDA-MB-231 was purchased from the ATCC and these breast cancer cell lines were tested to verify the absence of Mycoplasma contamination when cells were prepared. These breast cancer cell lines were authenticated by genetic profiling using polymorphic short tandem repeat loci (Promega, WI, USA).

### Western blotting

Western blotting was performed as previously described [16]. Cells were lysed in 1 × SDS sample buffer (50 mM Tris–HCl (pH 6.8), 2% SDS, 5% 2-mercaptoethanol, 0.1% BPB, 10% glycerol) and then boiled at 95 °C for 5 min. To examine NIK protein level, cells were treated with 10 μM MG132 (Peptide Institute, Inc., Osaka, Japan) for 4 h. To assess cIAP1 protein stability, cells were treated with 10 µg/mL cycloheximide (CHX) (Fujifilm Wako Pure Chemical Corporation). In addition, hypotonic buffer (10 mM HEPES—KOH (pH 7.9), 1.5 mM MgCl_2_, 10 mM KCl, Protease inhibitor cocktail, and 0.15 mM DTT) was used to fractionate nuclear lysates and cytoplasmic lysates. Then, 3 × SDS sample buffer was added to each lysate. The lysates were subjected to SDS-PAGE using Mini-PROTEAN TGX gels (Bio-Rad Laboratories Inc., CA, USA) and transferred to PVDF membranes (Millipore, Darmstadt, Germany). After blocking with Blocking One regent (NACALAI TESQUE, INC., Kyoto, Japan) for 1 h, these membranes were incubated with primary antibodies at 4 °C overnight. Then, these membranes were washed with Tris Buffered Saline with Tween^®^20 (Takara Bio Inc., Shiga, Japan) and anti-rabbit or mouse IgG HRP-linked antibody (cytiva, Tokyo, Japan) conjugated for 1 h at room temperature. Finally, the target proteins were detected using ImmobilonTM Western (Millipore) and Fusion Solo7S (Vilber-Lourmat, Seine-et-Marne, France). To strip these antibodies, these membranes were immersed in boiled water for 5 min. The quantification of protein production was calculated by the intrinsic software of Fusion Solo7S. The quantitative data of post-translational modified protein was normalized by each amount of non-modified protein and control protein (α-tubulin) data. Information regarding the primary antibodies and secondary antibodies is described in Additional file 1: Table S1.

### Protein stability analysis

To assess cIAP1 protein stability, cells were treated with 10 µg/mL cycloheximide (CHX) (Fujifilm Wako Pure Chemical Corporation) for 4, 8 and 12 h. Following the method of western blotting, cIAP1 protein levels were normalized by the amount of control protein (α-tubulin) at each time point. Then, relative cIAP1 protein levels were calculated based on a non-treated sample (0 h).

### Immunoprecipitation

Breast cancer cells were lysed in TNE buffer (20 mM Tris–HCl (pH 8.0), 150 mM NaCl, 1% NP-40, 2 mM EDTA, 25 mM NaF, 17.5 mM β-glycerophosphate, 1 mM Na_3_VO_4_, Protease inhibitor cocktail). The protein concentrations of the cell lysates were measured by the BCA method (Thermo Fisher Scientific, MA, USA). Then, each protein lysate (1 mg) was added to a NIK antibody (#4994, Cell Signaling Technology, MA, USA, 140 ng) or a rabbit IgG antibody (12–370, Millipore, 140 ng) and mixed with rotation overnight at 4 °C. After mixing, 20 µL of Protein A Sepharose 4 FF (GE Healthcare Japan, Tokyo, Japan) was added and mixed with rotation at 4 °C for 3 h. Subsequently, the supernatant was removed after centrifugation, and the Protein A Sepharose was washed five times with TNE Buffer. Finally, 1 × SDS sample buffer was added after removing the supernatant followed by heating at 95 °C for 5 min. This extract solution was used as the immunoprecipitation sample.

### RNA extraction and quantitative reverse transcription polymerase chain reaction (qRT-PCR)

Total RNA was extracted by QIAzol (Qiagen, CA, USA) according to the manufacturer’s protocol. After purification of the total RNA, RNA was quantified and checked the quality by NanoDrop One (Thermo Fisher Scientific, ND-ONE-W). RNA was reverse-transcribed into cDNA by the SuperScript™ First-Strand Synthesis System for RT-PCR (Thermo Fisher Scientific). Quantitative PCR was performed with Thunderbird SYBR qPCR mix (TOYOBO CO., LTD., Osaka, Japan) and the StepOnePlus real-time PCR system (Applied Biosystems, Foster, CA, USA). Quantification of the relative mRNA expression levels was performed by normalization to the level of β-actin RNA. The oligonucleotide sequences of the qRT-PCR primers are listed in Additional file 1: Table S2.

### Cell growth assay

Breast cancer cells (5×10^4^ cells/well) were plated on the 12-well plates and incubated overnight. After incubation, each cell was washed once with D-PBS(-) and incubated for 5 minutes in 0.25% Trypsin-EDTA (Thermo Fisher Scientific). Then the normal culture medium of RPMI-1640 was added to each well up to 1 mL. The cell number of each sample was calculated by loading the cell suspension medium into a hemocytometer.

### Soft agar assay

RPMI culture medium containing 0.3% agarose (Lonza, Basel, Switzerland) with LM05-shGFP or LM05-shNIK cells (4.0 × 10^4^ cells/well) over a bottom layer of 0.6% agarose in RPMI culture medium were plated in each well of a 6-well plate and cultured for 3 weeks. Colonies were fixed with 4% paraformaldehyde-PBS (Fujifilm Wako Pure Chemical Corporation) for 1 h and stained with 0.005% crystal violet solution (Fujifilm Wako Pure Chemical Corporation) for 30 min. After removing the overdyed region with Milli-Q water, the colony images were acquired with a digital camera (Nikon Corporation, Tokyo, Japan), and colony numbers were calculated with ImageJ software (National Institutes of Health, MD, USA).

### Boyden chamber assay

LM05-shGFP or LM05-shNIK cells (1.5 × 10^4^ cells/well) were plated on the lower chamber of 24-well plates and incubated overnight. The culture medium was replaced with Advanced RPMI-1640 medium (Thermo Fisher Scientific) supplemented with 1%(V/V) antibiotic–antimycotic (Thermo Fisher Scientific). After incubation for 48 h, the upper chamber of 8 μm pore size transwell inserts (Corning, NY, USA), which were placed with TIG-3 cells (1 × 10^4^ cells/well) in serum-free medium, was placed in the lower chamber. After incubation for 12 h, migrated cells at the lower surface of the membrane were fixed with 4% paraformaldehyde–PBS and stained with 0.005% crystal violet solution. Then margin liquid and cells on the upper surface of the membrane were removed with a cotton swab. Images were acquired with a BZ-X700 microscope (Keyence Corporation, Osaka, Japan) and analyzed using ImageJ software.

### Co-culture assay by transwell

TIG-3 cells (5 × 10^3^ cells/well) were plated on the lower chamber of 6-well plates and incubated overnight. After incubation, the upper chamber of 0.8 μm pore size transwell inserts (Corning), which were placed with LM05-shGFP or LM05-shNIK cells (1 × 10^4^ cells/well), were placed in the lower chamber. After incubation for 7 days, TIG-3 cells at the lower chamber were lysed in 1 × SDS sample buffer and boiled at 95 °C for 5 min, followed by western blotting.

### Immunofluorescence stain

Brest cancer cell lines and TIG-3 cells were fixed with 4% paraformaldehyde–PBS for 15 min and permeabilized with 0.1% Triton X-100-PBS (Fujifilm Wako Pure Chemical Corporation) for 15 min. After blocking with Blocking One regent for 1 h, cells were incubated with primary antibodies against α-SMA (#19245, Cell Signaling Technology, 1:500), Vimentin (V2258, Sigma-Aldrich Co., MO, USA, 1:200), NF-κB2 (#05–361, Merck, Darmstadt, Deutschland, 1:100), RelB (#4922, Cell Signaling Technology, 1:100) at 4 °C overnight. Then, cells were incubated with Hoechst 33342 (H3570, Invitrogen, MA, USA) and the secondary antibody conjugated Alexa Fluor 488 and 594 (A11001, A21207, Invitrogen, 1:500) for 1 h in room temperature. Images were acquired with a BZ-X700 microscope.

### Click reaction

Parental and LM05 cells were washed once with D-PBS(-) and incubated for 4 h in methionine-free RPMI-1640 (Sigma-Aldrich Co.) supplemented with 10% heat-inactivated FBS, GlutaMAX (Thermo Fisher Scientific), and 55 μg/mL L-cystine (Sigma-Aldrich Co.). Then, the media was replaced with media supplemented with 50 μmol/L L-homopropargyl glycine (HPG) (Cayman Chemical, MI, USA) for 24 h. To examine nascent NIK protein production, cells were treated with 50 μmol/L L-HPG and 10 μM MG132 for 4 h. After the treated cells were dissolved in lysis buffer (50 mM HEPES—KOH (pH 7.3), 150 mM NaCl, 0.1% NP-40, Protease inhibitor cocktail), nascent HPG proteins and biotin-PEG3-azide (Tokyo Chemical Industry Co. Tokyo, Japan) were chemically reacted overnight using the Click-iT Protein Reaction Buffer Kit (Thermo Fisher Scientific) at 4 °C according to the manufacturer's protocol. After the click reaction, the biotinylated proteins were resuspended in TNE buffer, and Dynabeads™ M-280 Streptavidin (Thermo Fisher Scientific) was added to the resuspended protein lysate. Then, the solution was incubated overnight at 4 °C using a rotary shaker. Streptavidin–biotin conjugates were washed five times with TNE buffer. After washing, the conjugates were resuspended in 1 × SDS sample buffer and eluted by boiling at 95 °C for 5 min, followed by western blotting.

### Animal experiments

Animal experiments were performed in compliance with the guidelines of the Institute for Laboratory Animal Research, National Cancer Center Research Institute (experimental number: T18-009) and the Animal Committee of Waseda University (Accession Numbers: WD19-058, 2019-A068, WD20-005, 2020-A067, WD21-082, 2021-A074). The tumor size reached the limits defined in these approved animal protocols (specifically, expected tumor weight did not exceed 10% of the mouse body weight or if the mice presented with body condition indicative of weight loss of > 20% of their initial weight). Female NOD.CB-17-*Prkdc*-scid/J mice (NOD-SCID, 5–6 weeks old, Charles River Laboratories Japan, Inc., Kanagawa, Japan) were used for the orthotopic xenograft and tail vein injection models. The methods for establishing the xenograft metastasis model and tail vein injection model and the performance of bioluminescence imaging were previously described [14]. For orthotopic xenografts, a total of 1.0 × 10^6^ cancer cells in 10 µl of D-PBS(-) were xenografted into the fourth fat pad of each mouse using a 28-gauge syringe (ITO CORPORATION, Shizuoka, Japan) after abdominal incision. These tumor volumes were calculated based on the formula of elliptical ball volume (V = 4/3 * π * A * B * C). A, B, and C are the lengths of all three semi-axes of the tumor. When the primary tumor volumes reached 300 mm^3^, they were resected under anesthesia with 2.5% isoflurane (Fujifilm Wako Pure Chemical Corporation). After the primary tumors were resected, mice were periodically monitored for metastasis formation using an in vivo imaging system (IVIS) -Lumina XRMS (Perkin-Elmer, Waltham, MA, USA) for 2 weeks. For the tail vein injection model, a total of 5.0 × 10^5^ cancer cells in 100 µl of D-PBS( −) were injected into the tail vein of each mouse using a 27-gauge needle. To monitor these xenograft mice, 200 µL of D-luciferin (15 mg/mL) (Gold Biotechnology, Inc., MO, USA) was injected intraperitoneally into each mouse, and bioluminescence imaging was performed with the IVIS every week. In addition, the bioluminescence of the lung metastatic tissue was measured ex vivo using the IVIS after intraperitoneal administration of 200 µL of D-luciferin to assess the metastatic potential of the cancer cells.

### Histochemical analyses

The dissected primary tumors and lung metastasis tissues from the orthotopic mouse model were fixed with 4% paraformaldehyde–PBS and embedded in paraffin. The paraffin-embedded human breast cancer cells with a breast tissue microarray (BC081116d and BC081116e, US Biomax, MD, USA) were baked for 2 h at 60 °C before proceeding with the following steps. The paraffin sections were deparaffinized and rehydrated in xylene (Fujifilm Wako Pure Chemical Corporation), a graded ethanol series (Fujifilm Wako Pure Chemical Corporation) that decreased stepwise from 100 to 50%, and distilled water.

For immunohistochemistry (IHC) analysis, antigen retrieval was performed in 10 mM citrate buffer (pH 6.0) (Fujifilm Wako Pure Chemical Corporation) at 120 °C or 95 °C for 20 min. After cooling to room temperature, the endogenous peroxidase activity was blocked with 3% H_2_O_2_ (Fujifilm Wako Pure Chemical Corporation) for 30 min. Then, these sections were incubated in 2.5% normal horse serum (Vector Laboratories, CA, USA) for 1 h at room temperature. The specimens were incubated with primary antibodies against α-SMA (A2547, Sigma-Aldrich Co., 1:100), Ki-67 (ab16667, Abcam, Cambridge, UK, 1:200), CAM5.2 (#349205, BD Bioscience, CA, USA, 1:1), NIK (HPA027269, Sigma-Aldrich Co., 1:50) and cIAP1 (ab108361, Abcam, 1:100) at 4 °C overnight. Then, the sections were incubated with the secondary antibody solutions and VECTASTAIN® ABC Reagent (Vector Laboratories) for 1 h at room temperature. The sections were stained with an ImmPACT® DAB EqV substrate kit (Vector Laboratories) followed by staining with hematoxylin (Polysciences Inc., PA, USA), dehydration and mounting. Images were acquired with a BZ-X700 microscope and analyzed using the image analysis application for BZ-X700 and ImageJ software.

For hematoxylin and eosin (HE) staining, deparaffinized and rehydrated sections were stained in Mayer's hematoxylin solution (Muto Pure Chemicals Co., Tokyo, Japan) for 10 min. Then, the sections were soaked in 0.1% saturated lithium carbonate at 37 °C for 5 min. After washing in distilled H_2_O, the sections were stained with eosin solution (Muto Pure Chemicals Co.) for 10 min. Then, the sections were immediately washed with 100% ethanol, 90% ethanol and xylene. Finally, the sections were mounted, and images were acquired with a BZ-X700 microscope.

For terminal deoxynucleotidyl transferase dUTP nick end labeling (TUNEL) staining, deparaffinized and rehydrated sections were stained with a TUNEL Assay Kit (ab206386, Abcam) according to the manufacturer’s protocols. After staining with hematoxylin, dehydration and mounting, images were acquired with a BZ-X700 microscope.

### RNA-seq analysis

Total RNA was extracted with QIAzol (Qiagen) according to the manufacturer’s protocol. After purification of the total RNA, the quantity and quality of the RNA were evaluated with a Nanodrop ND-1000 spectrophotometer (Thermo Fisher Scientific) and Agilent2100 Bioanalyzer (Agilent, CA, USA). cDNA libraries for RNA sequencing were established from total RNA using NEBNext Poly(A) mRNA Magnetic Isolation Module (New England Biolabs, MA, USA) to select poly-A mRNA followed by strand-specific library preparation using MGIEasy RNA Directional Library Prep Set V2.0 (MGITech Co. Shenzhen, China). Paired-end sequencing with a lead length of 150 bases was performed on a DNBSEQ-G400 (MGI tech) platform following the manufacturer’s instructions.

The raw sequence data (GSE182261: fastq files) were trimmed to remove adapter sequences by Trim-Galore (v0.6.4), and then, the trimmed read data were mapped on GRCh38/hg38 (GENCODE) using HISAT2 (v2.2.0). After the SAM files were converted to Bam files using SAMtool (v1.10), the gene expression FPKM was quantified by StringTie (v2.1.2). Then, read count matrices were generated for each gene by prepDE.py of StringTie. After removing low-expression genes with less than 10 counts per million (CPM), differentially expressed genes were identified using edgeR in R. Statistical cutoffs based on a p-value < 0.05 and a log fold change (Log2(FC)) ± 1 were used to filter differentially expressed genes (DEGs) between LM05-shGFP cells and LM05-shNIK cells. The z scores of these DEGs were calculated by gene filtering, and hierarchical clustering heatmaps were created using pheatmap in R (v3.6.2). For pathway analysis, the expression data were analyzed with gene set enrichment analysis (GSEA; https://www.gsea-msigdb.org/gsea/index.jsp) and ingenuity pathway analysis (IPA; https://digitalinsights.qiagen.com/products-overview/discovery-insights-portfolio/analysis-and-visualization/qiagen-ipa/).

### Expression and knockdown vector constructs

NIK, cIAP1 and HA tag-fused ubiquitin (HA-UB) expression vectors were previously produced [17, 18]. The coding region of HA-UB was transferred to the pMXs vector using Ligation High (Toyobo Co., Ltd., Osaka, Japan). shNIK knockdown vectors were previously produced [19]. All cDNA and shRNA sequences were confirmed by sequencing. Cloning primers and target sequences of shRNA are described in Additional file 1: Table S3.

### Retroviral packaging and infection

The production of the retrovirus using Plat-E cells was previously described [20]. Parent-shGFP, Parent-HA-UB, LM05-shNIK, LM05-shGFP, LM05-HA-UB, LM05-Venus, LM05-cIAP1 (human), and LM05-TAP-cIAP1 (murine) cells were established by retroviral infection of the pMXs-target gene cDNA-IRES-Puro^R^ overexpression vectors, pMMLV-BIRC2-IRES-Puro^R^ overexpression vectors (VB211123-1472ash, Kanagawa, VectorBuilder) or pSuper-target shRNA-Psv40-Puro^R^ knockdown vectors (OligoEngine, WA, USA) and selected with 3 µg/ml puromycin (Fujifilm Wako Pure Chemical Corporation). LM05-NIK rescue cells were established by retroviral infection of pMXs-NIK-Psv40-Neo^R^ overexpression vectors with LM05-shNIK no.2 cells and selected with 1 µg/ml G418 (Fujifilm Wako Pure Chemical Corporation).

### Statistical analysis

All statistical analyses were performed with GraphPad Prism 7 (GraphPad Software, CA, USA). Statistical results are presented as the mean ± SEM. Welch’s t-test and one-way or two-way ANOVA were employed for comparisons between two or three groups of data.

## Results

### The nuclear localization of NF-κB2 (p52) and RelB was facilitated in LM05 cells via NIK upregulation at the protein level.

**Fig. 1:**
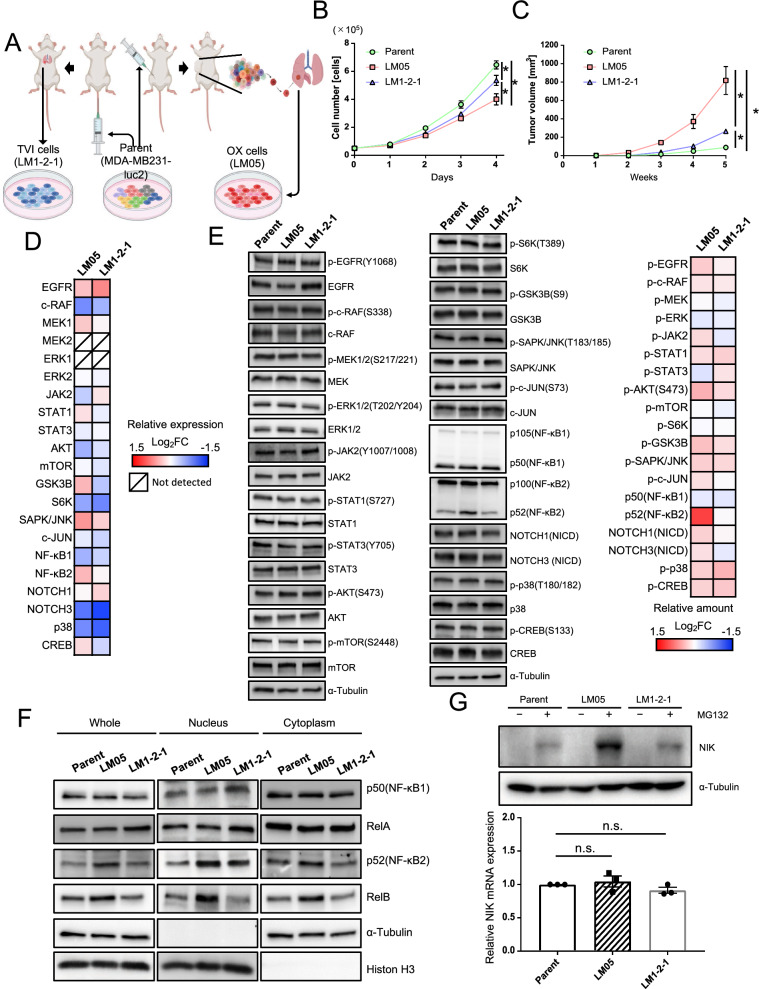
The nuclear localization of NF-κB2 (p52) and RelB was facilitated in LM05 cells via NIK upregulation at the protein level. **A** Schematic representation of the in vivo selection process using an orthotopic xenograft (OX) and tail vein injection (TVI). Luciferase-expressing MDA-MB-231 (parental cells) cells were transplanted into NOD-SCID mice by OX or TVI. Subsequently, lung metastatic cells were collected and established from lung tissue with metastases. These cells were reinjected into NOD-SCID mice using the same xenograft model to concentrate these cells with higher lung metastatic activity. **B** Cell growth curves of Parent, LM05 and LM1-2-1 cell lines on planar culture (n = 3, two-way ANOVA followed by Tukey’s multiple comparison test). **C** Representative tumor growth curves (lower, n = 4 per group, two-way ANOVA followed by Tukey’s multiple comparison test) of NOD-SCID mice orthotopically injected with parental, LM05 or LM1-2-1 cells at 0 week and 5 weeks. **D** Integrative signaling analysis of LM05 and LM1-2-1 cells was analyzed by microarray expression data. The microarray expression results were shown as a heatmap of the log_2_-fold change in each cell compared with the parental cells. **E**. Representative primary blots and heatmap of integrative signaling analysis by western blotting. The protein production data of western blotting were shown as a heatmap of the log_2_-fold change in each cell compared with the parental cells. **F** Western blotting analysis of NF-κB1 (p50), NF-κB2 (p100/52), RelA, RelB, α-Tubulin and Histone H3 in whole cells and the nuclear and cytoplasmic extracts of the parental, LM05, and LM1-2-1 cells. **G** Western blotting (upper) and qRT-PCR (lower, n = 3, one-way ANOVA followed by Tukey’s multiple comparison test) results of NIK protein and mRNA in the parental, LM05, and LM1-2-1 cells. For western blotting, all cell lines were either untreated or treated with MG132 (10 μM for 4 h). All data are representative of three independent experiments and are shown as the mean ± SEM. *n.s.* not significant. * P < 0.05

We previously established two lung metastatic breast cancer cell lines (LM05 and LM1-2-1) [14]. LM05 cells were established from MDA-MB-231 cells (parental cells) by two cycles of the generation of OX and subsequent extraction from the lung metastatic tissue (Fig. 1A). LM1-2-1 cells were established from parental cells by two cycles of TVI and subsequent extraction from the lung metastatic tissue (Fig. 1A). The cell proliferation of LM05 decreased compared with the parental cells and LM1-2–1 cells (Fig. 1B). Oh the other hand, LM05 cells had a high potential for tumor growth in the fat pads compared with the parental cells and LM1-2–1 cells (Fig. 1C) [14]. In previous studies, transcriptome analysis of these highly malignant cell lines was performed as a conventional research strategy [21–23]. In this study, considering the importance of translational and post-translational modifications in cancer malignancy, we investigated proteins that changed in the amount and/or modification after the translation process by comparison between western blotting and the microarray expression data to discover the factors that enhanced tumor growth. We employed LM1-2–1 cells as the comparison subject to search for specific factors in LM05 cells. The analysis results showed that the gene expression related to EGFR-MAPK (EGFR, c-RAF, MEK1/2, ERK1/2), AKT-mTOR (AKT, mTOR, GSK3B, S6K1), JAK-STAT (JAK2, STAT1/3) and, other pathways (JNK, NOTCH, CREB and so on) did not markedly change (|Log_2_(FC)|> 1) (Fig. 1D, Additional file 1: Table S4, S5, Additional file 2: Fig. S1). The protein production and post-translational modification did not markedly change, although the protein level of NF-κB2 (p52) increased (Log_2_(FC) > 1) (Fig. 1E, Additional file 1: Table S6). One of the NF-κB signaling activation markers is the nuclear translocation of the NF-κB family of proteins: p105/p50 (NF-κB1), p100/p52 (NF-κB2), RelA (p65), and RelB [24, 25]. Therefore, we investigated the nuclear translocation of the NF-κB family proteins by western blotting to evaluate the activity of NF-κB signaling in LM05 cells. Signaling analysis revealed that p50 and RelA in the nuclear fraction was unchanged in LM05 cells (Fig. 1F). On the other hand, NF-κB2 (p52) and RelB increased in nuclear of LM05 cells compared with the parental cells and LM1-2–1 cells (Fig. 1F, Additional file 2: Fig. S2). The nuclear translocation of p52 and RelB is attributed to the partial degradation of p100 to p52 [26, 27]. Since p52 and RelB are classified as part of the non-canonical NF-κB pathway, we examined the amounts of NF-kappa-β-inducing kinase (NIK), a key regulator of the non-canonical NF-κB pathway [28]. NIK is rapidly degraded by the ubiquitin–proteasome system after being ubiquitinated with the TRAF2-TRAF3-cIAP1/2 complex [29, 30]. Therefore, we investigated the amounts of NIK protein in the presence of a proteasome inhibitor, MG132. As a result, NIK increased at the protein level even though NIK mRNA expression did not significantly change in LM05 cells (Fig. 1G). These results indicated that nuclear localization of NF-κB2 (p52) and RelB was facilitated in LM05 cells via NIK upregulation at the protein level.

### NIK upregulation was induced by the suppression of nascent cIAP1 protein production

**Fig. 2:**
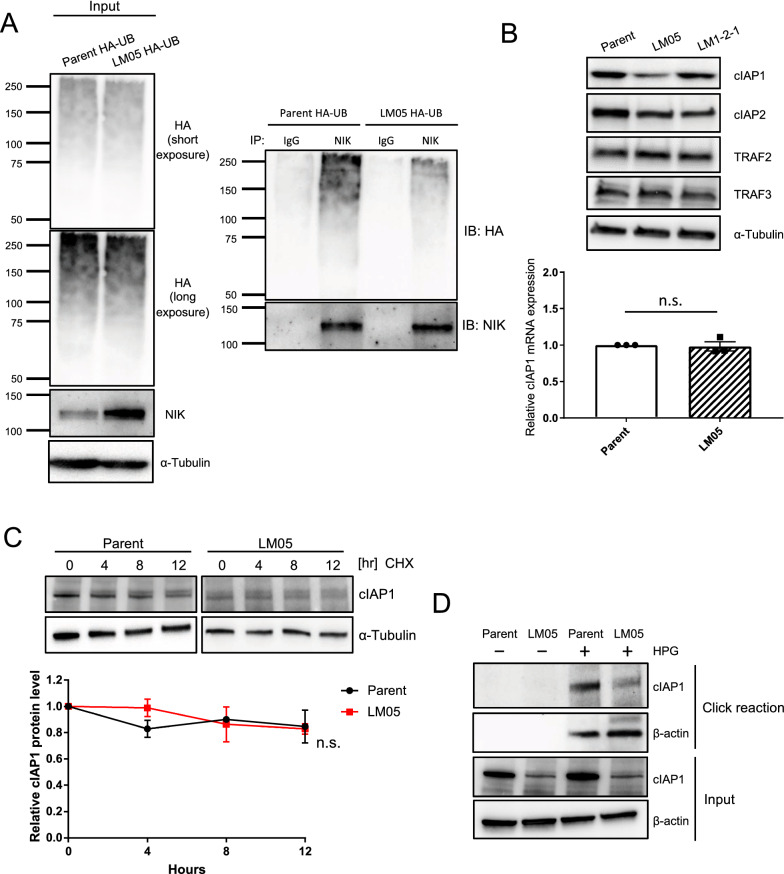
Downregulation of cIAP1 translation caused the upregulation of NIK protein. **A** Western blotting analysis of ubiquitinated NIK in HA-tagged ubiquitin (HA-UB)-expressing parental or LM05 cells after treatment with 10 μM MG132 for 4 h. The protein lysates of HA-UB expressed in parental or LM05 cells were immunoprecipitated with an anti-NIK antibody and then immunoblotted with an anti-HA antibody. **B** Representative western blotting (upper) of cIAP1, 2, TRAF2 and 3 in parental, LM05 and LM1-2–1 cells. qRT-PCR (lower, n = 3, Welch’s t-test) analysis of cIAP1 expression in parental and LM05 cells. **C** Assessment of cIAP1 protein stability in parental and LM05 cells treated with 10 µg/mL cycloheximide (CHX). Quantification of cIAP1 protein level normalized by α-Tubulin protein level in each time point (n = 3, two-way ANOVA followed by Bonferroni's multiple comparisons test). **D**. Assessment of nascent cIAP1 protein levels in parental and LM05 cells using a click reaction. Parental and LM05 cells were treated with 50 μM L-homopropargyl glycine (HPG) for 24 h. The nascent proteins labeled with HPG were conjugated to biotin using a click reaction. The biotinylated proteins were purified with streptavidin beads and subjected to western blotting. All data are representative of three independent experiments and are shown as the mean ± SEM. *n.s.* not significant

NIK is constantly degraded in the normal state by the TRAF2-TRAF3-cIAP1/2 complex in the ubiquitin–proteasome system [29, 30]. We examined the ubiquitination of NIK by immunoprecipitation with a NIK antibody. Ubiquitination of NIK decreased in LM05 cells compared with parental cells (Fig. 2A). Nest, we examined the amounts of TRAF2, TRAF3 and cIAP1/2 protein, which acts as degradation of NIK [29, 30]. From this result, the protein production of TRAF2 and 3 did not differ between parent, LM05 and LM1-2–1 cells (Fig. 2B). Although cIAP1 mRNA expression was unchanged compared to the parental and LM05 cells, western blotting analysis showed that cIAP1 decreased at the protein level (Fig. 2B). In addition, NIK upregulation in LM05 cells was suppressed by cIAP1 overexpression (Additional file 2: Fig. S3A). In the parent cell line, NIK production increased by cIAP1 knockdown (Additional file 2: Fig. S3B). Next, we evaluated the protein stability of cIAP1 by cycloheximide chase analysis to elucidate the downregulation mechanism of cIAP1. These results demonstrated that the cIAP1 reduction at the protein level was not ascribed to protein degradation in LM05 cells (Fig. 2C). In previous studies, it is established that nascent cIAP1 production was occasionally regulated by translational mechanisms [31–33]. Hence, we evaluated the production of the nascent cIAP1 protein pulse-labeled with L-homopropargylglycine (HPG) as a methionine analog conjugated to a biotin tag using a click reaction. The results showed that HPG-labeled cIAP1 protein production decreased in LM05 cells compared with parental cells (Fig. 2D). Furthermore, we also evaluated that the production of nascent NIK protein using the click reaction. This result displayed that the synthesis of nascent NIK protein did not differ between parent and LM05 cells (Additional file 2: Fig. S3C). All of these results suggested that the translational downregulation of cIAP1 caused post-translational stabilization of NIK in LM05 cells.

### Upregulation of NIK contributed to tumor growth in LM05 cells

**Fig. 3:**
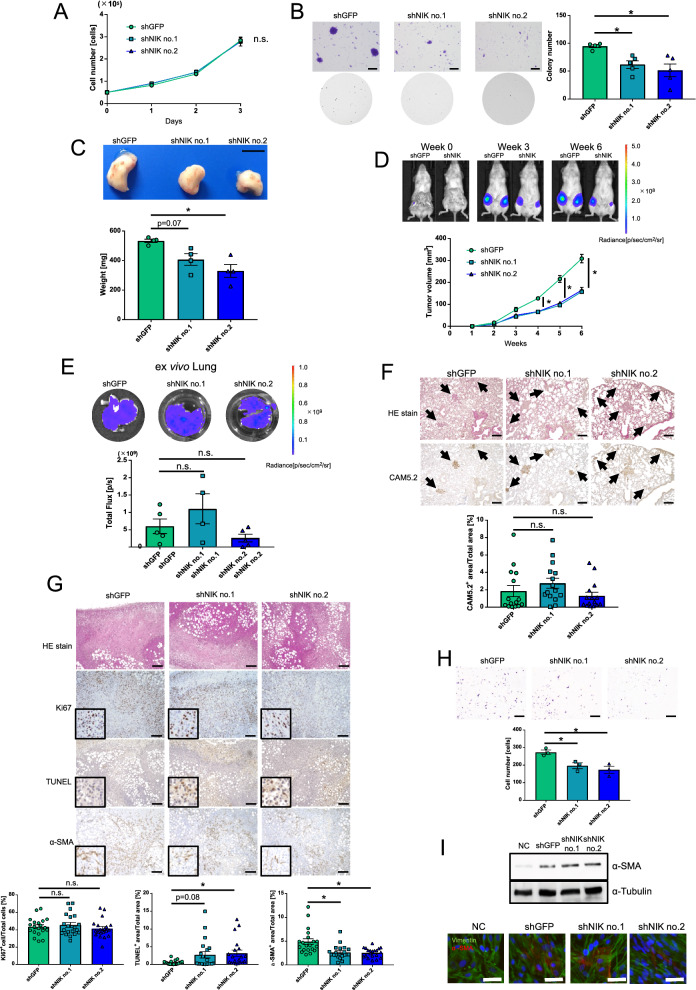
NIK upregulation facilitated inherent tumor growth but not the lung metastatic potential of LM05 cells.** A** Cell growth curves of LM05-shGFP, shNIK no.1 and shNIK no.2 cells on planar culture (n = 3, two-way ANOVA followed by Tukey’s multiple comparison test). **B** Representative images (upper) and quantification data (lower) (n = 5, one-way ANOVA followed by Tukey’s multiple comparison test) of the soft agar colony formation assay in LM05-shGFP, shNIK no.1 and shNIK no.2 cells. The scale bar is 5 mm. **C** Representative images of primary tumors (upper) and quantification data of the primary tumor weights (lower) (n = 4, one-way ANOVA followed by Tukey’s multiple comparison test) in LM05-shGFP, shNIK no.1 and shNIK no.2 cells. The scale bar is 1 cm. **D** Representative in vivo bioluminescent images of LM05-shGFP and shNIK no.2 cells (upper). Tumor growth curves (lower) (n = 6 per group, two-way ANOVA followed by Tukey’s multiple comparison test) of NOD-SCID mice orthotopically injected with LM05-shGFP, shNIK no.1 and shNIK no.2 cells. **E** Representative ex *vivo* bioluminescent images (upper) and quantification data of the lung metastasis tissue (lower) (one-way ANOVA followed by Tukey’s multiple comparison test) derived from NOD-SCID mice orthotopically injected with LM05-shGFP (n = 5), shNIK no.1 (n = 4) and shNIK no.2 cells (n = 5). **F** Representative HE and IHC staining images (upper) and quantification data (lower) (one-way ANOVA followed by Tukey’s multiple comparison test) of NIK protein production in lung metastasis tissue derived from NOD-SCID mice orthotopically injected with LM05-shGFP, shNIK no.1 and shNIK no.2 cells. The average CAM5.2-positive percentages were calculated from 5 fields of view from n = 4 individual lung metastasis slides. The scale bar is 100 μm. **G** Representative HE and IHC staining images (upper) and quantification data (lower) (one-way ANOVA followed by Tukey’s multiple comparison test) from the TUNEL assay and α-SMA protein production in primary tumor tissues derived from NOD-SCID mice orthotopically injected with LM05-shGFP, shNIK no.1 and shNIK no.2 cells. The average number of TUNEL- and α-SMA-positive cells were calculated from 5 fields of view from n = 4 individual primary tumor slides. The scale bar is 100 μm. **H** Representative images (upper) and quantification data (lower) (n = 5, one-way ANOVA followed by Tukey’s multiple comparison test) of the Boyden chamber assay with TIG-3 cells that were co-cultured with LM05-shGFP, shNIK no.1, and shNIK no.2 cells. The scale bar is 500 μm. **I** Western blotting (upper) and immunofluorescence staining (lower) of α-SMA expression in TIG-3 cells that were co-cultured with LM05-shGFP, shNIK no.1 and shNIK no.2 cells or no cells (NC). The scale bar is 50 μm. All data are shown as the mean ± SEM. *n.s.* not significant. * P < 0.05

To determine the contribution of NIK to cancer malignancy, we established NIK knockdown cells of LM05. NIK knockdown cells depressed NIK expression and nuclear localization of p52 and RelB (Additional file 2: Fig. S4A, B). We evaluated cell proliferation and anchorage-independent growth with a soft agar assay. NIK expression did not affect cell growth in planar culture (Fig. 3A), although the colony formation activity was decreased by NIK knockdown (Fig. 3B). Next, we investigated whether NIK upregulation contributes to the inherent high tumor growth and lung metastatic potential of LM05 cells, NIK knockdown cells were orthotopically injected into the mammary fat pads of NOD-SCID mice. NIK knockdown in LM05 cells significantly decreased the primary tumor weights and volumes (Fig. 3C and D). The reduction in tumor growth caused by NIK knockdown was partially rescued by recovering the ectopic expression of NIK (Additional file 2: Fig. S5A, B and C). In addition, we examined the effects of NIK knockdown on lung metastatic potential by performing a metastasis assay in an orthotopic xenograft model. To remove the inhibitory effects of tumor growth caused by NIK knockdown, we uniformly resected the primary tumors after they reached a size of 300 mm^3^. Under these conditions, NIK knockdown did not affect the potential of LM05 cells to form lung metastasis (Fig. 3E). IHC analysis of lung metastases using an anti-CAM5.2 antibody, a human-specific cytokeratin, indicated that no differences in the metastatic node areas between the control and NIK knockdown groups (Fig. 3F). These results suggested that NIK upregulation facilitated the inherent tumor growth but not the lung metastatic potential of LM05 cells.

Next, we performed immunohistochemical (IHC) staining of the primary tumor to investigate the role of NIK in the primary tumor. The probability of Ki67 positive cells (proliferation marker) didn’t change between the primary tumor of LM05-shGFP and LM05-shNIK (Fig. 3G). On the other hand, the number of TUNEL-positive regions (apoptosis marker) tended to increase in the primary tumor after NIK knockdown (Fig. 3G). The α-SMA-positive region (a cancer-associated fibroblast (CAF) marker) decreased in the primary tumors after NIK knockdown (Fig. 3G). Therefore, we utilized a co-culture system in a Boyden chamber to investigate the effects of NIK on the human fibroblast cell line TIG-3. Our results revealed that NIK knockdown suppressed the attraction of TIG-3 cells to the cancer cells in the co-culture system (Fig. 3H). On the other hand, α-SMA protein in TIG-3 cells did not change when co-cultured with control and NIK knockdown cells (Fig. 3I), indicating that NIK was involved in the attraction of fibroblasts but did not induce their activation to CAFs. These results suggest that the suppression of tumor growth caused by NIK knockdown is due to increased apoptosis of the tumor cells at least in part and decreased induction of CAFs.

### NIK knockdown suppressed cancer-inducing inflammatory signaling

**Fig. 4:**
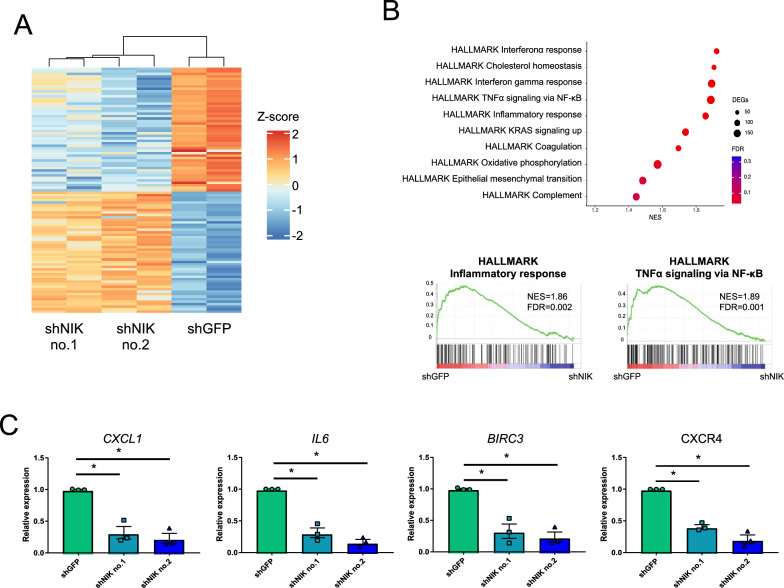
NIK regulated cancer-inducing inflammatory signaling in LM05 cells. **A** Heatmap of the DEGs from the LM05-shGFP, LM05-shNIK no.1 and LM05-shNIK no.2 RNA-seq data, each performed in duplicate. Hierarchical analysis of the heatmap was performed by the complete linkage method. **B** GSEA enrichment plot of the hallmark gene sets for the differentially expressed genes in LM05-shNIK cells compared with LM05-shGFP cells. **C** Validation of NIK signature gene expression related to tumor inflammation using qRT-PCR (lower) (n = 3, one-way ANOVA followed by Tukey’s multiple comparison test) in LM05-shGFP, shNIK no.1 and shNIK no.2 cells. All data are shown as the mean ± SEM. *n.s.* not significant. * P < 0.05

To determine the NIK-related pathway and genes that contribute to the phenotypes altered by NIK knockdown (Fig. 3B, C, D, G and H), we performed transcriptome analysis by RNA-seq. We extracted the differentially expressed genes (DEGs) between LM05-shGFP cells and LM05-shNIK cells using the following criteria: FDR < 0.05 (Benjamini–Hochberg method) and log fold change |(Log_2_(FC))|> 1 (Fig. 4A, Additional file 1: Table S7). To determine the NIK-related pathways, we performed gene set enrichment analysis (GSEA) and ingenuity pathway analysis (IPA). GSEA using the MSigDB Hallmark gene set collection revealed that these downregulated genes with NIK knockdown were significantly enriched for pathways related to inflammation, interferon response, and TNFα signaling via NF-κB (Fig. 4B, Additional file 1: Table S8). Similarly, pathways related to the interferon response and TNF secretion were suppressed in NIK knockdown cells using Gene Ontology gene sets (Additional file 1: Table S9). Indeed, NIK knockdown decreased the expression of *IL6* and *CXCL1*, which are important for the attraction of fibroblasts (Fig. 4C) [34, 35]. *BIRC3* expression, which contributes to apoptosis resistance, markedly decreased by NIK knockdown (Fig. 4C) [36, 37]. These results suggested that NIK knockdown caused the reduction in anti-apoptotic gene expression and fibroblast-attracted cytokines and chemokines, inherently resulting in decreased tumor growth.

### NIK protein production increased in malignant breast cancer tissue

**Fig. 5:**
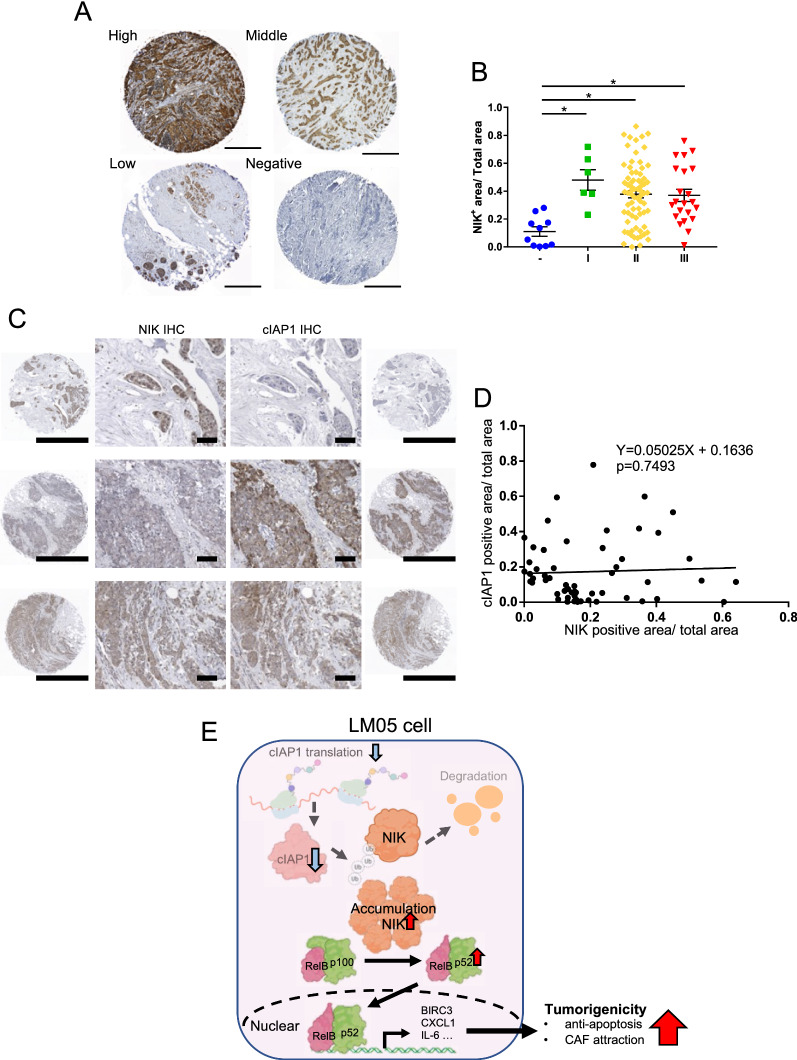
NIK protein production increased in malignant breast cancer tissue. **A** Representative IHC staining images of NIK protein production in normal breast tissue and breast tumors. The scale bar is 500 μm. **B** Quantification data of the NIK IHC staining images in normal breast tissue and breast tumors ((n = 10 normal, n = 6 stage I, n = 72 stage II, and n = 22 stage III); one-way ANOVA followed by Tukey’s multiple comparison test). **C** Representative IHC staining images of NIK and cIAP1 protein production in normal breast tissue and breast tumors of consecutive cuts. The scale bar of the low-power field is 1 mm and the scale bar of the high-power field is 100 μm. **D** Representative scatter plot of NIK and cIAP1 positive percentage in IHC stain tissue. The p-value and equation were calculated by Linear regulation analysis (n = 55). **E** The graphical summary indicates that the abnormal accumulation of NIK, due to reduced translation of cIAP1, enhances tumor growth by promoting an inflammatory cancer microenvironment. All data are shown as the mean ± SEM. *n.s.* not significant. * P < 0.05

We investigated the relationship between NIK production and breast cancer malignancy in clinical samples. We characterized NIK production by IHC staining in a tissue microarray from breast cancer patients (Fig. 5A). Firstly, we could also observe the difference in NIK protein production between control and NIK knockdown xenograft tumor tissues using the NIK antibody (Additional file 2: Fig. S6A). IHC analysis showed that NIK protein production significantly increased in stage I-III patients compared with normal breast tissues (Fig. 5B, Additional file 1: Table S10). NIK protein production did not correlate with the expression scores of breast cancer marker genes such as ER, PR, and HER2 (Additional file 2: Fig. S7A, B and C). From these results, NIK has the potential to be used as a diagnostic marker across other breast cancer subtypes. On the other hand, the cIAP1 protein production slightly increased in these tumor tissues, but the cIAP1 protein level didn’t differ statistically between normal and tumor tissue (Additional file 2: Fig. S8). We investigated the correlation of NIK and cIAP1 protein production in clinical breast cancer tissues of consecutive cuts. In NIK or cIAP1 expression tissues (each positive percentage > 10%), no significant negative correlation between NIK and cIAP1 protein production was observed in clinical breast cancer tissues (Fig. 5C, D). However, it is notable that 72% of tissues exhibited the NIK^high^/cIAP1^low^ or NIK^low^/cIAP1^high^ pattern (NIK + percentage > 10% and cIAP1 + percentage < 10%, or NIK + percentage < 10% and cIAP1 + percentage > 10%) (Fig. 5C, Additional file 1: Table S11). Therefore, the reduction of cIAP1 contributed to the NIK upregulation in a subset of primary human breast cancer tissues.

## Discussion

NIK expression increased in the several breast cancer cell lines and multiple myeloma cell lines [38–40]. In breast cancer, basal-like subtype cell lines have shown increased NIK mRNA expression by epigenetic dysregulation of the NIK gene [40, 41]. Certain multiple myeloma cell lines have genetic defects in TRAF2/3 and cIAP1/2 [38, 39], which degrade NIK protein. This study indicated that the reduction of translation of cIAP1 increased the amount of NIK protein. This was a novel cause of the aberrant accumulation of NIK in the breast cancer cell line (Fig. 5E).

The activation of the non-canonical NF-κB pathway by NIK upregulation contributes to the enhancement of cell proliferation and maintenance of cancer stemness properties via the NOTCH pathway, especially in basal-like breast cancer cell lines [19, 40]. Although claudin-low cell lines exhibit high levels of NF-κB activation with increased NIK mRNA expression [40, 41], the function of NIK in tumor malignancy and metastasis has not been sufficiently investigated. Our results suggested that NIK upregulation contributed to tumor growth by attracting CAFs and partial anti-apoptosis potency through regulation of the expression of genes related to inflammatory responses in LM05 cells, which is classified as the claudin-low subtype (Fig. 5E). Therefore, our present study proposed that there were novel function and aberrant accumulation mechanism of NIK in breast cancer that differs from the conventional research.

From our results, a reason for the reduction of tumor growth by NIK knockdown was the expansion of the apoptotic area in the primary tumor. CAFs contribute to the enhancement of tumor growth by promoting the antiapoptotic potential and chemoresistance of cancer cells [42, 43]. It was possible that NIK knockdown suppressed CAFs production, leading to the decline in the antiapoptotic potency in tumors. CAFs secreted CXCL12/SDF-1, which contributes to tumor growth and anti-apoptosis in cancer cells via CXCR4 [44, 45]. Indeed, the expression of *CXCR4* decreased in NIK knockdown cells. Thus, it is possible that the positive feedback through this paracrine mechanism is repressed by NIK knockdown. Therefore, these data suggested that NIK plays an important role in the interaction with stromal cells in the cancer microenvironment.

NIK is crucial for the maintenance of various tissue functions, including the immune system, bone formation, the kidneys, the liver, glucose homeostasis and hematopoiesis [46]. Then, abnormal NIK activation has been implicated in a variety of autoimmune diseases, such as systemic lupus erythematosus, acute kidney injury and cancer [46]. Recently, exploratory studies of NIK inhibitors have expanded [46]. Owing to NIK crystallographic data, novel NIK inhibitors have been developed based on structure–activity relationships and docking simulations [47, 48]. These studies have demonstrated that NIK inhibitors are therapeutically effective in certain mouse models of inflammatory hepatic diseases and systemic lupus erythematosus [49, 50]. As another example, mangiferin, which is a natural compound with NIK inhibitory activity, has been reported to suppress tumor growth and metastatic potential in melanoma cell lines [51]. However, the efficacy of these NIK inhibitors against other types of cancer is limited; thus, it would be necessary to investigate their potential broadly. In contrast, inactivating NIK mutations have been shown to cause immunodeficiencies, such as decreased numbers of mature B cells and T cells and their functional impairment [52]. NIK plays an important role in antitumor immunity by regulating metabolism in cytotoxic CD8^+^ T cells [53]. Therefore, NIK inhibitors need to be developed with a better molecular understanding so as not to interfere with their homeostatic role in the body.

Our experimental results indicated that NIK knockdown critically affected tumor growth rather than the potential to form lung metastases in LM05 cells. From this result, we hypothesized that NIK may play an important role in tumor growth at the mammary gland. To address this hypothesis, we evaluated the proliferative potential of parental and LM05 cells in lung tissue via tail vein injection. The results showed that no significant difference in the proliferative potential of the lung tissues between the parental and LM05 cells (Additional file 2: Fig. S9). LM05 cells were repeatedly xenografted into the mammary gland and grown at this site. Indeed, TWEAK and RANKL, ligands of the non-canonical NF-κB pathway, are expressed and have important functions in mammary gland development and tumor malignancy [54–57]. In short, LM05 cells may be more susceptible to the accumulation of NIK proteins by these ligands in breast tissue. Meanwhile, we did not identify the genes that contribute to the enhanced potential of LM05 cells to metastasize to the lungs. For this issue, we attempted to extract a group of genes that upregulated in LM05 cells compared to the parental strain but not downregulated after NIK knockdown. The group included known lung metastasis regulatory genes such as *IL13RA2*, *TNS1*, and *EMP1* [22, 58, 59] (Additional file 2: Fig. S10). Further studies of those genes may reveal the molecular mechanism of NIK-independent metastasis enhancement in LM05 cells.

This present study has limitations. The IHC experimental result of NIK and cIAP1 suggested that the reduction of cIAP1 and other bioprocesses might contribute to the NIK upregulation in human breast cancer tissue. We conjectured that the downregulation of TRAF2 and TRAF3 may contribute to NIK upregulation as other bioprocesses. In previous research, TRAF2 and TRAF3 are also the degradation factor of NIK protein [29, 30] and these proteins are frequently deleted and inactive mutated in several cancer types [60]. Hence, further research is needed to elucidate the mechanism of NIK upregulation in clinical breast cancer tissue.

## Conclusion

In conclusion, our results demonstrated a novel role and the attribution of NIK upregulation in a high malignancy breast cancer cell line. In addition, the amount of NIK protein increased in tumor tissues compared to normal tissues; thus, NIK has the potential to be a diagnostic marker of breast cancer. Elucidating the functions and regulatory mechanisms of NIK can lead to a deeper understanding of NIK as a potential biomarker or therapeutic target in breast cancer.

## Supplementary Information

**Additional file 1**: **Table S1** List of antibody used in this study. **Table S2** List of RT-qPCR primer used in this research. **Table S3** List of primer and shRNA sequence used in this research. **Table S4** Gene expression results of microarray data (Comparison with parent cells: log2FC). **Table S5** Gene expression results of RT-qPCR (Comparison with parent cells: log2FC). **Table S6** Protein expression results of western blot (Comparison with parent cell: log2FC). **Table S7** DEGs list of RNA-seq analysis (criteria: FDR <0.05 (Benjamini-Hochberg method) and log fold change |Log2(FC)|>1). **Table S8** GSEA result of the RNA-seq data using Hallmark gene set. **Table S9** GSEA result of the RNA-seq data using GO gene set. **Table S10** NIK IHC result of breast cancer tissue microarray. **Table S11** NIK and cIAP1 IHC result of breast cancer tissue microarray (consecutive cuts).SP:sample peel.

**Additional file 2**: **Figure. S1**. The validation of the microarray analysis result by RT-qPCR for target genes of the signal analysis. **Figure. S2**. The validation of the western blotting data by immunofluorescence staining of NF-κB2 (p100/52) and RelB. **Figure. S3**. The alteration of NIK protein production by cIAP1 ectopic expression and knockdown control exhibit negative correlation in LM05 and Parent cells. **Figure. S4**. NIK knockdown cell liens were decreased in NIK expression and nuclear localization of p52 and RelB. **Figure. S5**. NIK ectopic expression partially rescued the reduction in tumorigenicity induced by NIK knockdown. **Figure. S6**. The anti-NIK antibody is valuable for IHC to detect NIK protein. **Figure. S7**. NIK expression was not correlated with PR, ER, or HER2 scores. **Figure. S8**. cIAP1 protein level didn’t differ significantly between normal and tumor tissue. **Figure. S9**. The TVI model showed that the lung metastatic potential of LM05 cells was not enhanced compared with parental cells. **Figure. S10**. Some known lung metastasis-promoting genes are highly expressed in the LM05 cell line independent of NIK knockdown.

## Data Availability

RNA-seq data were submitted to the NCBI GEO database under accession number GSE182261. The microarray expression data of the breast cancer cells that can generate lung metastases were obtained from our previous research [14]. These expression data and other supporting data of this research are available from the corresponding authors upon reasonable request.

## Abbreviations

NF-κB: Nuclear Factor-Kappa B
NIK: NF-kappa-β-Inducing Kinase
cIAP1: Cellular Inhibitor of Apoptosis Protein 1
TNBC: Triple-Negative Breast Cancer
OX: Orthotopic Xenograft
TVI: Tail Vein Injection
