# Supplementary material for: Aberrant accumulation of NIK promotes tumor growth by dysregulating translation and post-translational modifications in breast cancer
Source: Cancer Cell Int. 2023 Apr 1;23:57. doi: 10.1186/s12935-023-02904-y (PMC10067241; doi:10.1186/s12935-023-02904-y)
Supplement: Supplementary file 2 — Additional file 2: Figure. S1. The validation of the microarray analysis result by RT-qPCR for target genes of the signal analysis. Figure. S2. The validation of the western blotting data by immunofluorescence staining of NF-κB2 (p100/52) and RelB. Figure. S3. The alteration of NIK protein production by cIAP1 ectopic expression and knockdown control exhibit negative correlation in LM05 and Parent cells. Figure. S4. NIK knockdown cell liens were decreased in NIK expression and nuclear localization of p52 and RelB. Figure. S5. NIK ectopic expression partially rescued the reduction in tumorigenicity induced by NIK knockdown. Figure. S6. The anti-NIK antibody is valuable for IHC to detect NIK protein. Figure. S7. NIK expression was not correlated with PR, ER, or HER2 scores. Figure. S8. cIAP1 protein level didn’t differ significantly between normal and tumor tissue. Figure. S9. The TVI model showed that the lung metastatic potential of LM05 cells was not enhanced compared with parental cells. Figure. S10. Some known lung metastasis-promoting genes are highly expressed in the LM05 cell line independent of NIK knockdown. [file 12935_2023_2904_MOESM2_ESM.pdf]

Additional file 2

Figure. S 1.

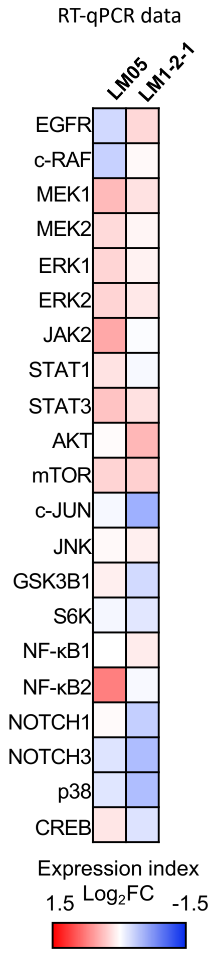

The validation of the microarray analysis result by RT-qPCR for target genes of the signal analysis.

These target genes expression of the signal analysis was analyzed by RT-qPCR. The expression results are shown as a heatmap of the $\log_2$-fold change in each cell compared with the parental cells.

Figure. S 2.

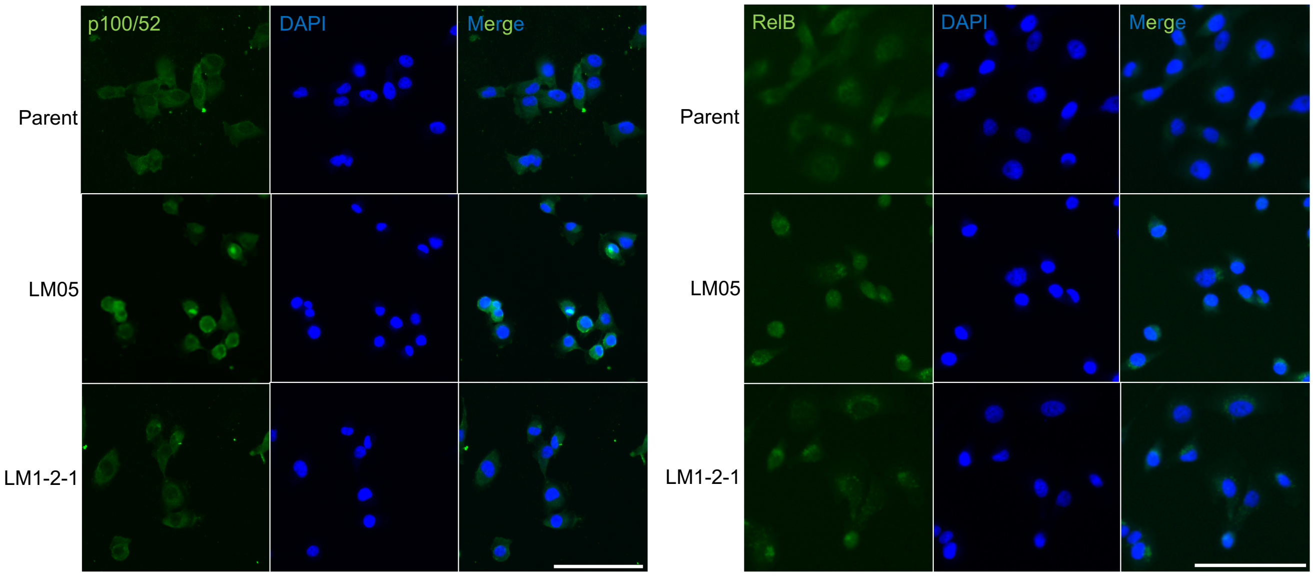

The validation of the western blotting data by immunofluorescence staining of NF-κB2 (p100/52) and RelB.

Representative immunofluorescence staining images of NF-κB2 (p100/52) and RelB in Parent, LM05, and LM1-2-1 cells. The scale bar is 100 μm.

Figure. S 3.

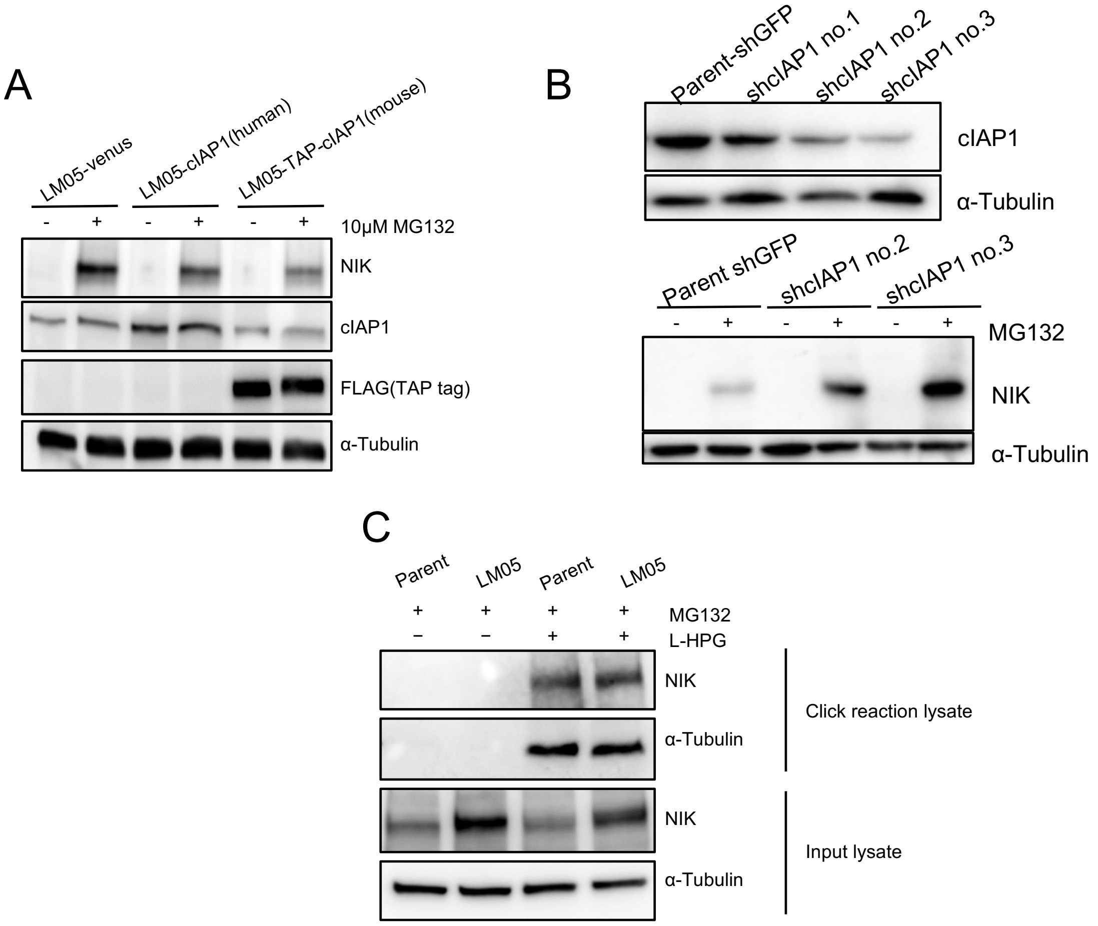

The alteration of NIK protein production by cIAP1 ectopic expression and knockdown control exhibit negative correlation in LM05 and Parent cells.

(A) Western blotting analysis of NIK protein production in parental, LM05-Venus, LM05-and LM05-cIAP1 (human) and LM05-TAP-cIAP1 (mouse) cells. For western blotting, all cell lines were either untreated or treated with MG132 (10 μM for 4 hr). **(B)** Representative Western blots of cIAP1, NIK and α-Tubulin in control (Parent-shGFP) and cIAP1 knockdown cell lines (Parent-shcIAP1 no.2, 3). For western blotting of NIK, all cell lines were either untreated or treated with MG132 (10 μM for 4 hr). **(C)** Western blotting analysis of nascent NIK protein in parental, LM05 cells. For western blotting, all cell lines were untreated or treated with MG132 (10 μM for 4 hr)

and L-homopropargyl glycine (HPG) (50 μM for 4 hr). The nascent proteins labeled with HPG were conjugated to biotin using a click reaction. The biotinylated proteins were purified with streptavidin beads and subjected to western blotting. All data are representative of three independent experiments.

Figure. S 4.

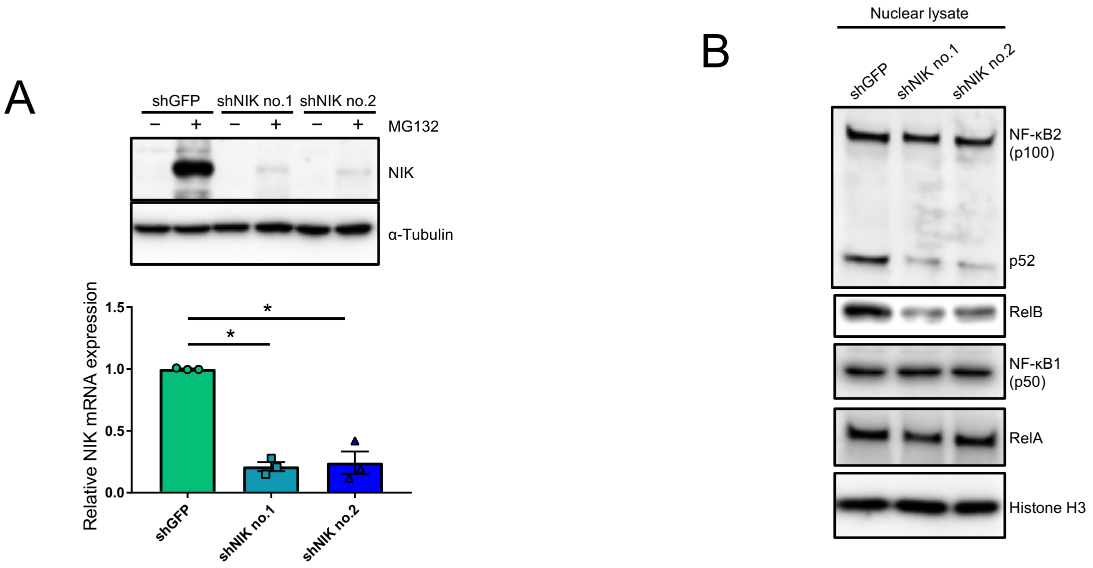

NIK knockdown cell liens were decreased in NIK expression and nuclear localization of p52 and RelB.

(A) Validation of NIK knockdown efficiency via Western blot (upper) and qRT-PCR (lower, n=3, one-way ANOVA followed by Tukey's multiple comparison test) analyses in LM05-shGFP, shNIK no.1 and shNIK no.2 cells. For western blotting, all cell lines were either untreated or treated with MG132 (10 μM for 4 hr). **(B)** Western blotting analysis of NF-κB1 (p50), NF-κB2 (p100/52), RelA and RelB in the nuclear extracts of LM05-shGFP, shNIK no.1 and shNIK no.2 cells. All data are representative of three independent experiments and are shown as the mean±SEM. NS, not significant. * $P<0.05$.

Figure. S 5.

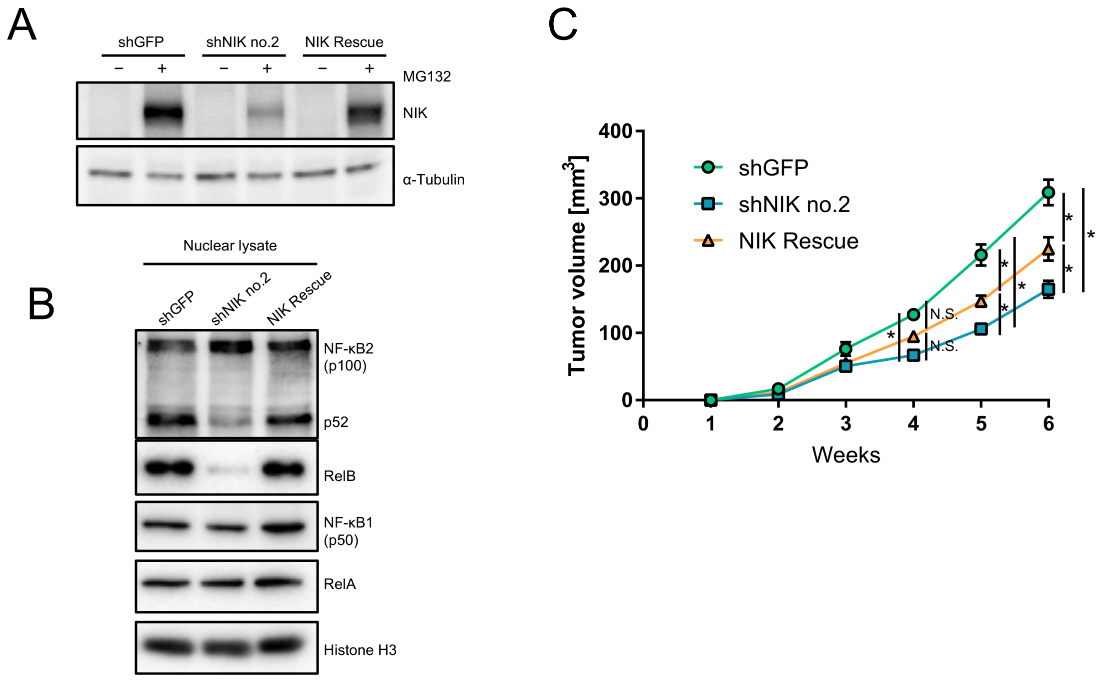

NIK ectopic expression partially rescued the reduction in tumorigenicity induced by NIK knockdown.

(A) Validation of the NIK rescue efficiency via Western blot analysis in LM05-shGFP, shNIK no.2 and NIK rescue cells. For western blotting, all cell lines were either untreated or treated with MG132 (10 μM for 4 hr). **(B)** Western blotting analysis of NF-κB1 (p50), NF-κB2 (p100/52), RelA and RelB in the nuclear extracts of LM05-shGFP, shNIK no.2 and NIK rescue cells. **(C)** Tumor growth curves (two-way ANOVA followed by Tukey's multiple comparison test) of NOD-SCID mice orthotopically injected with LM05-shGFP (n=6), shNIK no.2 (n=6) and NIK (n=4) rescue cells. All data are representative of three independent experiments and are shown as the mean±SEM. NS, not significant. * P<0.05.

Figure. S 6.

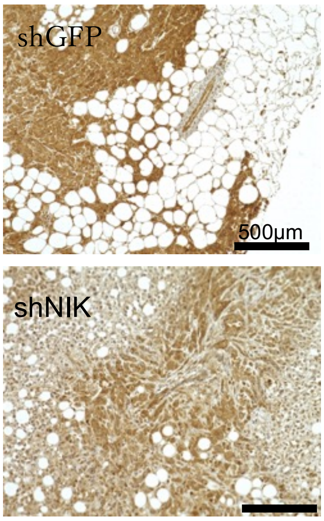

The anti-NIK antibody is valuable for IHCt to detect NIK protein.

Representative IHC staining images of NIK protein production in primary tumor tissues of orthotopically xenograft model: LM05-shGFP, shNIK cells. The antibody of IHC is the NIK antibody.

Figure. S 7.

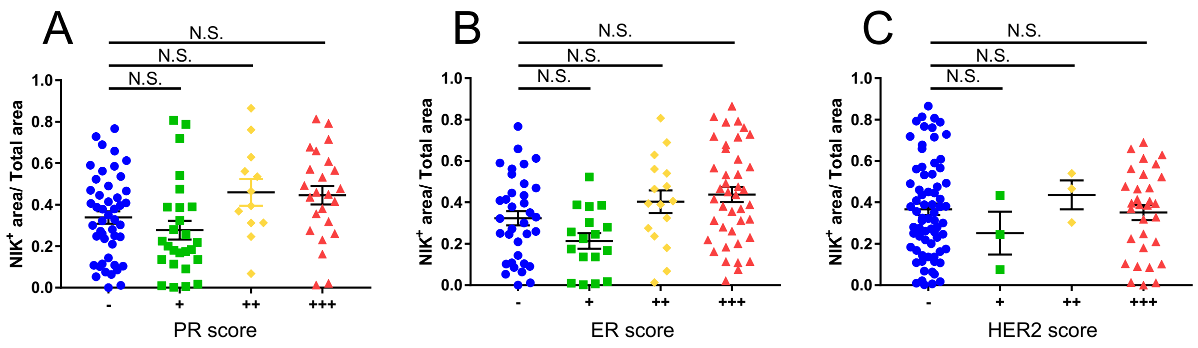

NIK expression was not correlated with PR, ER, or HER2 scores.

(A) Quantification data of the NIK IHC staining images of normal breast tissue and breast tumors for each PR score (n=47 - (negative), n=26 +, n=12 ++, and n=24 +++; one-way ANOVA followed by Tukey's multiple comparison test). **(B)** Quantification data of the NIK IHC staining images of normal breast tissue and breast tumors for each ER score (n=35 - (negative), n=17 +, n=26 ++, and n=41 +++; one-way ANOVA followed by Tukey's multiple comparison test). **(C)** Quantification data of the NIK IHC staining images of normal breast tissue and breast tumor for each HER2 score (n=72 - (negative), n=3 +, n=3 ++, and n=31 +++; one-way ANOVA followed by Tukey's multiple comparison test).

Figure. S 8.

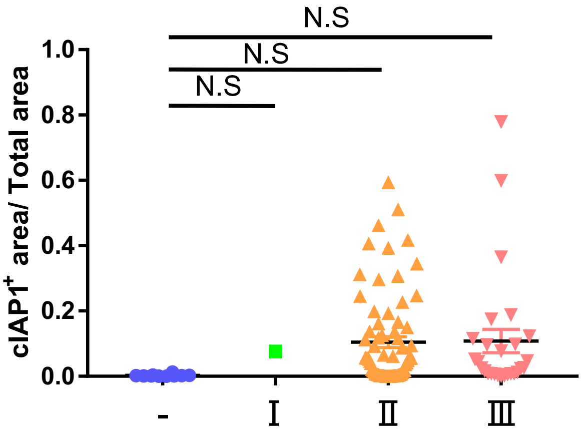

cIAP1 protein level didn't differ significantly between normal and tumor tissue.

Quantification data of the cIAP1 IHC staining images in normal breast tissue and breast tumors ((n=10 normal, n=1 stage I, n=72 stage II, and n=27 stage III); one-way ANOVA followed by Tukey's multiple comparison test).

Figure. S 9.

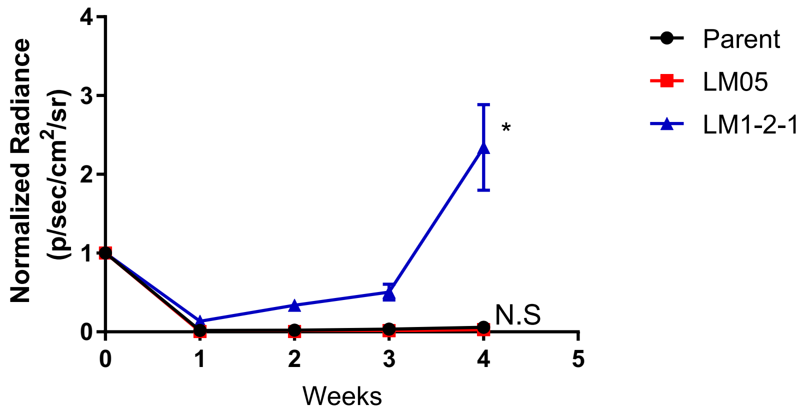

The TVI model showed that the lung metastatic potential of LM05 cells was not enhanced compared with parental cells.

Representative *in vivo* bioluminescent images (left) and quantification data of lung metastases (right) (one-way ANOVA followed by Tukey's multiple comparison test) derived from NOD-SCID mouse tail vein injection with parental, LM05, or LM1-2-1 cells (n=5).

Figure. S 10.

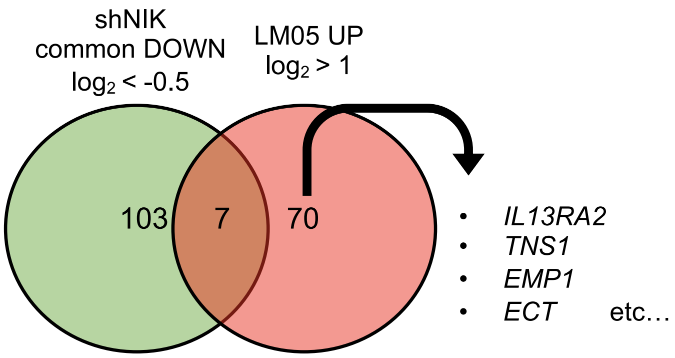

Some known lung metastasis-promoting genes are highly expressed in the LM05 cell line independent of NIK knockdown.

Venn diagram of the upregulated genes in LM05 cells based on previous microarray expression data and the downregulated genes found in the NIK KD cell line based on the RNA-seq data from this study. Expression of the known metastasis-promoting genes *IL13RA2*, *TNS1*, and *EMP1* is independent of NIK knockdown.
